# Adopting clinical genomics: a systematic review of genomic literacy among physicians in cancer care

**DOI:** 10.1186/s12920-018-0337-y

**Published:** 2018-02-13

**Authors:** Vu T. Dung Ha, Julie Frizzo-Barker, Peter Chow-White

**Affiliations:** Simon Fraser University, K8666, Shrum Science Building, 8888 University Drive, Burnaby, BC V5A 1S6 Canada

**Keywords:** Genomic literacy, Clinical genomics education, Genomic services, Oncology, Primary care physicians, Applied cancer genomics, Health education, Physicians, Genomic technologies, Genomic sciences

## Abstract

**Background:**

This article investigates the genomic knowledge of oncology care physicians in the adoption of clinical genomics. We apply Rogers’ knowledge framework from his diffusion of innovation theory to identify three types of knowledge in the process of translation and adoption: awareness, how-to, and principles knowledge. The objectives of this systematic review are to: (1) examine the level of knowledge among physicians in clinical cancer genomics, and (2) identify potential interventions or strategies for development of genomic education for oncology practice.

**Methods:**

We follow the PRIMSA statement protocol and conduct a search of five relevant electronic databases. Our review focuses on: (1) genomic knowledge of oncogenomics or genomic services in oncology practices among physicians, and (2) interventions or strategies to provide genomic education of oncogenomics for physicians.

**Results:**

We include twenty-one studies in our analysis. Nine focus on interventions to provide genomic education for cancer care. Overall, physicians’ knowledge of oncogenomics among the three types is limited. The genomic literacy of physicians vary by their provider specialty, location, years of practice, and the type of genomic services. The three distinctions of knowledge offer a sophisticated and helpful tool to design effective strategies and interventions to provide genomic education for cancer treatment. In the nine educational intervention studies, the main intervention outcomes are changes in awareness, referral rates, genomic confidence, and genomic knowledge.

**Conclusion:**

Rogers’ diffusion of innovation model allows us to differentiate three types of knowledge in the development and adoption of clinical genomics. This analytical lens can inform potential avenues to design more effective strategies and interventions to provide genomic education for oncology practice. We identified and synthesized a dearth of high quality studies that can inform the most effective educational outcomes of these interventions. Future research should attend to improving applications of genomic services in clinical practices, along with organizational change engendered by genomics in oncology practice.

## Background

Clinical genomic testing is becoming an integral part of medical care for oncology practice. For example, in clinical genomic trials for cancer, oncologists collaborate with genome scientists and other medical practitioners to analyze and evaluate meanings of genomic sequencing data for potential cancer treatments, thus bridging the clinical and research settings together [1]. Clinical genomics for cancer have the potential to revolutionize oncology practices into “more flexible, networked research arrangements, and towards using individual patients as model systems for asking biological questions” [2]. The genomic structure of each individual is different and genetic alterations vary from tumor to tumor. As a result of new genomic sequencing technologies, medical practitioners can diagnose, analyze, and treat cancer on the basis of an individual patient’s genome composition. However, one of the major challenges in this process is knowledge translation of genomic services into clinical care. The rapid expansion in genomic science and biomedical innovations produces novel knowledge and information that can generate uncertainty in the clinic and cast doubt among clinicians in terms of how to interpret and apply genomic data into clinical practice [3, 4]. A useful step in meeting this challenge is to understand the current level of knowledge in cancer clinical genomics among physicians.

Knowledge translation of genetics or genomic technologies into clinical practice is a topic that attracts the attention of researchers worldwide. A number of scholars have conducted systematic review studies on the barriers and strategies for the adoption of genomic services: for instance, systematic review studies by Suther and Goodson [5], Scheuner et al. [6], and Mikat-Stevens et al. [7] provide good examples of reporting challenges to the adoption of genetics services by primary care physicians. In a 2008 study, Scheuner et al. [6] identified several barriers to the delivery of genomic medicine in clinical care including inadequate genomic knowledge in primary care workforce, little awareness about genetics/genomics among consumers, and lack of high quality studies assessing clinical outcomes of genomic medicine. Nearly a decade later, this lack of genomic knowledge among primary care practitioners persists. In a recent study on perceived barriers to genetic services, Mikat-Stevens et al. [7] point out that deficits in physicians’ genomic knowledge, skills, and confidence are one of the main challenges to the integration of genetic services into clinical practices. To overcome these barriers, a group of researchers from the United Kingdom (U.K.) conducted a systematic review of educational interventions on clinical genetics for primary care physicians [8]. The U.K. review identifies eleven studies on genetics educational interventions that improved both knowledge and confidence of practitioners following the educational programmes. Yet, the authors find little evidence on changes in practice. Hence, the authors point out the need for future educational interventions studies to target more on changes in practice. While these studies contribute an important method for systematic review in perceived barriers and potential strategies to integrate genomic services, the scope of their review is broad and inclusive of all primary care specialties. Yet, genomic literacy and the importance of genomic testing vary by each provider specialty. In this systematic review, we focus solely on the genomic literacy of physicians on the front lines of cancer treatment. In other words, our review highlights physicians’ level of knowledge towards oncogenomics, as well as interventions to provide genomic education for oncology care. As such, the added values of our systematic review to this emerging field of genomic services delivery is an up-to-date systematic review of genomic knowledge among physicians in oncology care and potential strategies for physicians to improve their clinical cancer genomics literacy.

This study investigates the level of knowledge about genomics, known as “genomic literacy,” amongst physicians as captured in the biomedical literature over the last twenty years. The National Human Genome Research Institute at the National Institute of Health defines genomic literacy as the understanding of what a genome is, how genomic science works, and its affordances and limitations, applications, and impacts on society [9]. We employ Rogers’ knowledge translation theory “diffusion of innovations” [10] to identify and characterize different types of knowledge in the translation process of clinical genomics. Rogers identifies three types of knowledge building: awareness, how-to, and principles-based knowledge. We adapt these for the clinical genomics context. Awareness knowledge refers to having general knowledge or perception of oncogenomics and genomic services. How-to knowledge refers to practical knowledge about the application of oncogenomics and genomic services into oncology practice. Principles-based knowledge pertains to an understanding of the underlying theoretical principles of oncogenomics. Most studies tend to focus on levels of knowledge, such as high or low. While descriptive and instructive, high/low is a narrow framework that provides little insight into why an innovation may or may not be adopted. The three distinctions of knowledge provide a sophisticated and potentially insightful analysis into types of physicians’ literacy of clinical cancer genomics. Rogers’ knowledge framework, therefore, offers a tool to identify shortcomings in knowledge and knowledge translation of clinical genomics. The findings can then inform possible avenues to design effective strategies and interventions to provide genomic education for oncology practice.

The objectives of this systematic review are to (1) examine the level of knowledge among physicians in clinical cancer genomics and (2) identify potential interventions or strategies for the development of genomic education for oncology practice. The overall goal of this systematic review is to provide a holistic and insightful view of the current state of genomic knowledge among physicians in addressing the benefits, risks, and affordances in genomic research and technology.

## Methods

### Search strategy

**Fig. 1:**
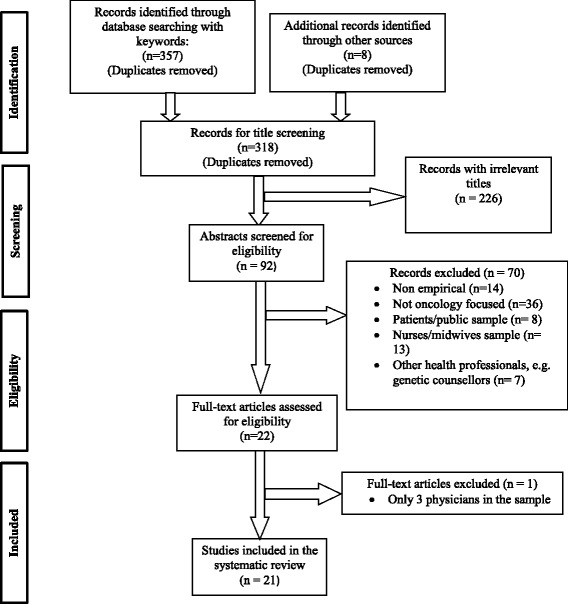
PRISMA flow chart

There are four steps in our review method: (1) data collection, (2) data screening and exclusion, (3) data inclusion, and (4) data analysis. We summarized our reviewing process in a PRISMA flowchart (Fig. 1). First, we identified and collected the articles by searching the following databases: Medline, Cumulative Index to Nursing and Allied Health Literature (CINAHL) Complete, Cochrane Central Register for Controlled Trials, Education Resources Information Center (ERIC), and PsychInfo. Our search covered the time period between January 2003 and July 2017. The rationale for the chosen date is that 2003 marked the completion of the Human Genome Project, which was a significant breakthrough for genetic and genomic research in medical science. In our search strategy, we looked for these terms in the abstract only: genetic education or genetic knowledge or genomic education or genomic knowledge and cancer or oncology. These searches identified 357 articles in total including 104 articles from Medline, 82 from CINAHL, 4 from Cochrane, 17 from ERIC, and 150 from PsychInfo. We removed duplicate papers. We also reviewed reference lists from the selected papers for potentially relevant articles with eight additional articles meeting the criteria after review.

### Selection of eligible articles

Our final inclusion criterion for this study was as follows: scholarly articles published in English, in peer-reviewed journals that reported on: (1) genomic knowledge of oncogenomics or genomic services in oncology practices among physicians, and (2) interventions or strategies to provide genomic education of oncogenomics for physicians. The research team reviewed the titles of the resulting 318 articles to assess inclusion eligibility based on the relevancy of the title to the field of genomic knowledge and genomic education. We excluded 226 records due to their irrelevant titles in relation to our systematic review topic. For example, we excluded articles that discussed biomedical research of genomic medicine or other social aspects of genomic medicine such as electronic health records of personalized genomic information, the dilemmas of disclosing incidental findings, clinical utility and validity of genomic information, the consumer satisfaction of genetic health services, genetic discrimination, the social and emotional challenges of genetic diseases on doctors, patients and families genetics education for the public, or internet resources in medical genetics.

Next, the reviewers assessed the remaining 92 articles based on the target population, provider specialty, and method eligibilities. Our target population included only doctors, clinicians, physicians, oncologists, and general practitioners. As a result, we excluded 28 articles because they did not include physician sample. Instead, they targeted patients or public samples (*n* = 8), nurses and midwives samples (*n* = 13), or other health professionals such as health educators and genetic counselors (*n* = 7). We excluded another 36 articles that did not focus on cancer or oncology care. We also excluded 14 articles that were not empirical. In addition, we excluded one article because it only contained three physicians in its study sample. For the final step, we conducted a full-text assessment of the remaining 22 articles. After cleaning the data according to our inclusion criteria, the total population included 21 articles.

### Data extraction and analysis

We used NVivo 10 research software to systematically review and code the included studies. The reviewers developed a coding scheme and ensured that all themes were mutually exclusive and exhaustive. For each node, we included several sub-themes to list the potential patterns each article may carry. Relevant data were identified from each article based on two themes: genomic knowledge and educational interventions. The research team then used the protocol outlined by the Centre for Reviews and Dissemination [11] to extract and enter data from reviewed studies in two tables outlining genomic knowledge and educational interventions. We extracted data from studies that focus on genomic knowledge (Table 1) and organized them in these following themes: (1) study, year, and country that produced the study, (2) methodology, (3) sample, (4) types of cancer (if available), (5) awareness knowledge, (6) how-to and principles knowledge, and (7) limitations of the study. For studies focusing on educational interventions (Table 2), we extracted data as follows: (1) study, year, and country produced the study, (2) aim, (3) methods, (4) participants, (5) intervention, (6) main outcomes of the intervention, and (7) limitations. In the process of coding the articles, we also identified a common theme among six studies that discuss factors associated with ordering, referring, or using genomic sequencing. We extracted the data and organized them into themes and studies (Table 3).

**Table 1:** Studies examining type(s) of genomic knowledge among physicians

Study, year, country	Method	Sample	Type of cancer	Awareness Knowledge	How-to Knowledge	Principles Knowledge	Limitations
Bellcross et al. (2011) United States [13]	Survey (response rate = 48%)	1500 physicians responded to 2007 DocStyles national survey	Breast-ovarian cancer	87% of respondents were aware of BRCA testing but only 25% reported having ordered at least one test in the past year	N/A	Only 19% correctly selected all of the increased-risk and none of the low-risk scenarios as an indication for BRCA testing. 45% chose at least one low-risk scenario	Sample may not be truly representative of primary care providers in US
Chow-White et al. (2017) Canada [1]	Survey (response rate = 52.5%)	31 medical oncologists (MOs) involved in a cancer clinical genomics trial	Not specified	N/A	67.8% of MOs somewhat and strongly agree that they feel confident to communicate genomic results to their patients. 58.1% of the MOs felt more confident making treatment decisions after becoming informed about patients’ genome.	MOs acknowledged they had little knowledge about never genomic technologies (50%) and whole genome sequencing process (41%). MOs located in urban/suburban areas tend to have higher genomic literacy than those located in rural areas.	Knowledge was self-reported Low number of respondents
Dressler et al. (2014) United States [14]	Survey	94 medical, surgical, and hematologic oncologists practicing in academic and community settings in North Carolina	Breast and Colorectal cancer	In one area, clinicians were not well aware with the term ‘pharmacogenomics’	37% of oncologists were comfortable in interpreting PGx test results. The use for breast cancer PGx testing vary: 100% for HER2 testing, 97% for Breast Oncotype Dx test, and 32% for CYP2D6 testing. Only 26% used Oncotype Dx for colon cancer.	Few oncologists were comfortable with their PGx knowledge (33%). Oncologists with less than 10 years of practice (48%) reported being more comfortable with their PGx knowledge than those with more than 10 years practice (21%).	Small sample no indication of required size
Freedman et al. (2003) United States [18]	15-min survey (response rate = 71%)	1251 physicians from 8 specialties	Not specified	N/A	Only 29% of physicians reported feeling qualified to provide genetic counselling for cancer susceptibility. Oncologists (84%) considered themselves more qualified to recommend genetic testing than primary care physicians (40%), and tertiary care physicians (57%)	N/A	Study measurement was self-reported
Gingras et al. (2016) Multi-countries [16]	28-item survey	215 physicians, of which 88% were MOs, practicing in Europe (70%) and working in academic institutions (66%)	Breast cancer	N/A	38% had used tumor genome sequencing for their breast cancer patients at least once in the past	21% reported low confidence in their genomic knowledge.	Small sample no indication of required size Article structure not well elucidated
Gray et al. (2014) United States [21]	Cross-sectional survey (response rate = 61%)	160 physicians including medical oncologists, surgeons, radiation oncologists	Not specified	N/A	N/A	22% reported low confidence in their genomic knowledge	Small sample size Study measurement was self-reported
Gray et al. (2016) United States [20]	Surveys (response rate = 92%) and Interviews	27 oncologists	Lung + Colorectal cancer	N/A	Oncologists had extensive experience ordering somatic tests (median = 100/year) but little experience ordering germline cancer predisposition tests per year (median = 2/year).	N/A	Small number of survey respondents Survey measures were self-reported
Koil et al. (2003) United States [19]	Survey (response rate = 25%)	214 physicians in Ohio’s tri-state region	Breast cancer	N/A	51% of the respondents reported having ever refereed for an indication of hereditary breast cancer. Rural-practice physicians were less likely to have ever referred for breast cancer testing than urban- and suburban-practice physicians combined	N/A	Low response rate Overall response bias for physicians with greater interest and experience in hereditary breast cancer
Marzuillo et al. (2013) Italy [17]	Survey (response rate = 69.6%)	1079 physicians	Breast + Colorectal cancer	N/A	Few physicians in the sample had either referred patients for predictive genetic testing for breast (10%) or colorectal cancer (4.7%) in the previous 2 years.	42.8% of the sample answered correctly all three knowledge questions about BRCA1/2 testing. 16.9% answered correctly about APC testing.	High percentage of non-responders Information about specialties was not adequate for meaningful comparisons
Wideroff et al. (2003, 2005) United States [12, 15]	15-min questionnaire (response rate = 71%)	1251 physicians (820 in primary care, 431 in selected subspecialties)	Breast+ ovarian+ Colorectal cancer	37.5% was aware of paternal inheritance of BRCA 1/2 mutations and 33.8% was aware that these mutations occur in < 10% of breast cancer patients	31.2% physicians in the sample, including 30.6% in primary care and 33.4% in tertiary care, had ordered cancer susceptibility tests (CSTs) or referred patients elsewhere for risk assessment or testing	One third of the sample accurately responded that less than 10% of breast cancer patients carry BRCA1/2 mutations. Only 13.1% accurately identified HNPCC gene penetrance as > 50%. OBGY, physicians, oncologists, and general surgeons were significantly more likely to respond correctly to breast/ovarian cancer questions, as were gastroenterologists to the HNPCC question	Ambiguous term for commercial availability. Small specialty subgroups

**Table 2:** Educational interventions to improve physicians' genomic literacy

Study, year, country	Aim	Methods	Participants	Intervention	Main outcomes	Limitations
Bethea et al. (2008) United Kingdom [26]	To determine the impact of genetic outreach education and support to primary care on practitioner’s confidence and competence in dealing with familial cancers	Quasi-experimental design: Longitudinal intervention study + Pre- and post- intervention knowledge survey	217 GPs and practice nurses from both rural and urban areas in England completed both pre- and post- intervention survey: 29 from intervention group, 188 from control group	In 2 areas, genetic educational outreach was provided to 10 randomly selected practices comprising two sessions on familial cancer and one on other genetic conditions	Respondents in the intervention group reported more confidence in dealing with issues related to management of patient queries around bowel cancer, knowing the relevant family history to collect, and making basic assessment of risk	Low response in pre- and post- intervention surveys from the intervention groups
Blazer et al. (2004) United States [22]	To determine the effects of a cancer genetics education program (CGEP) on clinical knowledge and practice	Quasi-experimental design: Longitudinal intervention study + Pre- and post- intervention knowledge survey + Practice impact surveys one year after the intervention	710 clinicians participated the program and completed the pre- and post- intervention survey. 69 out of 114 eligible annual conference participants sampled completed the practice impact surveys	The CGEP comprises: cancer genetics curriculum incorporating key elements published by American Society of Clinical Oncologists + six annual full day conferences covering in depth a specific topic in clinical cancer genetics	Respondents showed a 40% average increase in specific cancer genetics knowledge. Respondents to the practice impact survey reported that they used course information and materials to counsel and refer patients for hereditary cancer risk assessment (77%), shared course information with other clinicians (83%), and wanted additional cancer genetics education (80%).	Low response in the practice impact survey to produce convincing evidence on the long-term impact of the intervention
Blazer et al. (2011) United States [23]	To assess the impact of a multi-modal interdisciplinary course in genetic cancer risk assessment (GCRA) and research collaboration for community-based clinicians	Quasi-experimental design: Longitudinal intervention study + Pre- and post- intervention knowledge survey	131 participants (48 physicians, 41 advanced-practice nurses and 42 genetic counselors) from community settings across the U.S	The course was delivered in three phases: distance didactic learning, face-to-face training and 12 months of Web-based professional development activities to support integration of skills into practice. Cancer genetics knowledge, skills, professional self-efficacy and practice changes were measured at baseline, immediate- and 14-months-post course.	Knowledge, skills and self-efficacy scores were significantly different between practice disciplines; however, post scores increased significantly overall and for each discipline (*p* < .001). Fourteen-month practice outcomes reflect significant increases in provision of GCRA services (p = .018), dissemination of cancer prevention information (*p* = .005) and high-risk screening recommendations (*p* = .004) to patients, patient enrollment in research (*p* = .013), and educational outreach about GCRA (*p* = .003).	The majority of course participants had training in oncology or genetics; consequently, generalizability of the findings and adaptability to more primary care audiences is uncertain. Addition validation is needed with a more diverse set of clinicians
Hill et al. (2015) Multi-countries [24]	To increase childhood eye cancer retinoblastoma genetics knowledge by designing an interactive workshop for clinicians in Kenya	Quasi-experimental design: Longitudinal intervention study + Pre- and post- intervention knowledge survey + One-year post-workshop knowledge test	The workshop was conducted at the 2013 Kenyan National Retinoblastoma Strategy meeting. Knowledge was assessed by standardized test pre- and post- the workshop, and genetic counseling skills and confidence by questionnaire. One year after the workshop, the participants were asked to take a knowledge survey again	55 physicians attended the workshop, of whom 38 granted permission for use of their tests and permission to re-test after a year. 12 participants took the one-year post-workshop survey	Knowledge increased significantly post-workshop from 72% to 80%, driven by increased knowledge of retinoblastoma causative genetics. One-year post-workshop, participant knowledge had returned to baseline (72%), indicating that knowledge retention requires more frequent reinforcement. Participants reported feeling more confident discussing genetics with patients, and had integrated more genetic counseling into patient interactions.	Low response in the one-year post-workshop knowledge test
Houwink et al. (2014) Netherlands [28]	To examine sustained effects of online genetics education	Randomized controlled trial: a parallel-group, pre- and post-retention (6-month follow up) controlled group intervention trial was conducted, with repeated measurements using validated questionnaires	80 Dutch GPs responded to participate in the study. Two groups of 40 participants were estimated to be sufficient to detect a medium-to-large effect with a power of 90% and a significance level of 5%. Eighty Dutch GP volunteers were randomly assigned to the intervention or the control group. 24 from intervention group and 20 from control group completed the study	2-h online genetics education course: Genetics e-learning Continuing Professional Development (CPD) module, aiming at improving general practitioners’ (GPs’) knowledge about oncogenetics, Randomized controlled trial was also conducted to evaluate the outcomes at the first two levels of the Kirkpatrick framework (satisfaction, learning and behavior).	Satisfaction with the module was high, with the three item’s scores in the range 4.1–4.3 (5-point scale) and a global score of 7.9 (10-point scale). Knowledge gains post test and at retention test were 0.055 (*P* < 0.05) and 0.079 (*P* < 0.01), respectively, with moderate effect sizes (0.27 and 0.31, respectively). The participants appreciated applicability in daily practice of knowledge aspects (item scores 3.3–3.8, five-point scale), but scores on self-reported identification of disease, referral to a specialist and knowledge about the possibilities/limitations of genetic testing were near neutral (2.7–2.8, five-point scale).	Small sample size due to large attrition rate
Houwink et al. (2014) Netherlands [27]	To assess the effectiveness of oncogenetics training in general practitioners’ consultation skills	Blinded, randomized controlled trial (RCT) with parallel repeated measurements of intervention (received education) and control group, pre- and post-retention, with another 3-month follow up.	56 randomized Dutch GPs (38 intervention, 18 control group) completed the entire procedure, and 32 were lost to follow-up due to lack of time or sickness	4-h face-to-face training covering oncogenetic consultation skills	Key consultation skills significantly and substantially improved; regression coefficients after intervention were equivalent to 0.34 and 0.28 at 3-month follow-up, indicating a moderate effect size. Satisfaction and perceived applicability of newly learned skills were highly scored.	High number of non-responders, high attrition rates, especially in control group
Houwink et al. (2015) Netherlands [25]	To build effective educational modules on oncogenetics, in order to assess long-term increase in genetic consultation skills (1-year-follow-up) and interest in and satisfaction with a supportive website on genetics among GPs	Mixed-methods: Online questionnaire (effects of online and live training) + the pop-up questionnaire (appreciation of the website) + referral rates by GPs from the clinical genetics centres in the northern and southern parts of the Netherlands + website visitor analytics	Assessment 1. 168 GPs responded to an email invitation and were randomly assigned to an intervention or control group, evaluating the G-eCPD module (*n* = 80) or the live module (*n* = 88). Assessment 3: 38 website visitors completed the online questionnaire	Three oncogenetics modules were developed: an online Continuing Professional Development (G-eCPD) module, a live genetic CPD module, and a “GP and genetics” website (huisartsengenetica.nl) providing further genetics information applicable in daily practice. Three assessments to evaluate the effectiveness (1-year follow-up) of the oncogenetic modules were designed: 1.An online questionnaire on self-reported genetic competencies and changes in referral behaviour, 2. Referral rates from GPs to clinical genetics centres and 3.Satisfaction questionnaire and visitor count analytics of supportive genetics website.	Participants of these online and live training reported being more aware of genetic problems long term; this was reported by 29 GPs (69%) and 46 GPs (92%) participating in the G-eCPD and live module evaluation studies, respectively (Chisquare test, *p* < 0.005). One year later, 68% of the respondents attending the live training reported that they more frequently referred patients to the clinical genetics centres, compared to 29% of those who attended the online oncogenetics training However, the clinical genetics centres reported no significant change in referral numbers one year after the training. Website visitor numbers increased, as did satisfaction, reflected in a 7.7 and 8.1 (out of 10) global rating of the website (by G-eCPD and live module participants, respectively).	Long-term genetic consultation skills was self-reported Low number of respondents participated in the online questionnaire and the pop up questionnaire
Wilson et al. (2006) United Kingdom [30]	To evaluate the effectiveness of the intervention in improving GP confidence in managing patients concerned about genetic risk of breast cancer.	Cluster randomized controlled trial: multifaceted decision-supported intervention with intervention and control group, pre- and post- intervention questionnaires to both groups	All eligible practices (*n* = 86) were randomized, 57 (230 GPs) to the intervention group and 29 (116 GPs) to the control group. The total number of intervention and control GPs changed over the course of the trial because of natural turnover. Practices.	Multifaceted system which (i) built on a national IT initiative to assist GPs in interpreting and applying the new national guidelines for cancer genetics, including an explanation of the rationale underlying the risk stratification; (ii) contained elements to facilitate its use in general practice (e.g. information leaflets, contact email link); and (iii) complementary educational sessions which aimed to provide insight and skills training relating to breast cancer genetics.	No statistically significant differences were observed between intervention and control arms in the primary or secondary outcomes. A possible effect of the intervention on the proportion of referred patients who were at elevated risk could not be discounted. Only a small proportion of intervention GPs attended the educational session, were aware or the software, or made use of it in practice.	Small sample size, low statistical power Confidence in managing patient was self-reported measure
Westwood et al. (2012) United Kingdom [29]	To examine whether primary care genetic-led genetics education improves both non-cancer and cancer referral rates, and primary care-led genetics clinics improve the patient pathway.	Cluster-randomized factorial trial: educational intervention to receive case scenario based seminar (intervention) or not (control), and clinic location intervention includes referred patients a primary (intervention) or secondary (control)care genetic counsellor (GC)led appointment.	73 general practices in the south of England	A seminar was designed to include four case scenarios (cystic fibrosis, Huntington’s disease, breast cancer, and a child with dysmorphic features referred for diagnosis); referral access details; and the local and national cancer family history referral guidelines (breast, breast, and ovarian, ovarian, colorectal, and colorectal with other sites). An NHS intranet secure website, with access to cancer referral guidelines and a referral form, was developed.	Eighty-nine and 68 referrals made by 36 intervention and 37 control practices respectively. There was a trend towards an overall higher referral rate among educated GPs (referral rate ratio [RRR] 1.34) and they made more appropriate cancer referrals (RRR 2.36). 83% of GC-managed appointments met the 18-week referral to treatment, NHS target.	Inadequate sample size to demonstrate anything other than very large changes in clinic attendance rates, and in particular not adequate to demonstrate narrow equivalence limits.

**Table 3:** Factors for adopting cancer genomics services by category

Factors associated with ordering, referring or using genomic sequencing	Studies
Increases in knowledge, skills, and education	
Higher genomic confidence	Gray et al. (2014) [21]
Adequate knowledge of the professional use of predictive testing for breast cancer	Marzuillo et al. (2013) [17]
Increasing continuing medical education (CME)	Marzuillo et al. (2013) [17]
Feeling qualified to recommend cancer susceptibility tests (CSTs)	Wideroff et al. (2003) [15]
Having more than 25% of time allocated to research	Gingras et al. (2016) [16]
Receiving CSTs educational materials	Wideroff et al. (2003) [15]
Geographical factors	
Presence of genetic testing laboratories locally	Marzuillo et al. (2013) [17]
Practice location in the Northeast	Wideroff et al. (2003) [15]
Working in Asia	Gingras et al. (2016) [16]
Practice location in the urban and suburban areas	Koil et al. (2003) [19]
Patient interests, requests, and health records	
Family/patient history of cancer	Koil et al. (2003) [19]
Patient interests	Koil et al. (2003) [19]
Patient requests for genetic testing	Mazuillo et al. (2013) [17]
Professional guidelines	
Endorsement of genomic tests by American Society of Clinical Oncologist (ASCO) professional guidelines	Dressler et al. (2014) [14]
Having institutional guidelines for molecular sequencing	Gingras et al. (2016) [16]
Clinical utility and effectiveness of cancer genomics	
Evidence-based studies demonstrating safety and efficacy of the test	Dressler et al. (2014) [14]
Prospective clinical trials confirming association of testing with outcome	Dressler et al. (2014) [14]

## Results

Twelve of the 21 studies we analyzed reported on the general level of genomic knowledge amongst physicians. Table 1 summarizes the findings on the awareness, how-to, and principles-based knowledge reported in these 12 studies.

### Awareness knowledge of clinical genomics in oncology care

As the volume and scope of genomic information increases in health care contexts, practitioners will require basic genomic literacy in order to analyze, understand, interpret, and apply genomic information into clinical decisions. Only three out of 21 reviewed studies reported on awareness knowledge of physicians of genomics in cancer care. Our systematic review covered a broad timeframe (January 2003 – July 2017) as we were curious to identify whether the levels of genomic literacy changed over the time period. Our study did indeed show that there was an increase in awareness of *BRCA 1/2* mutations and testing between a study published in 2005 and another published in 2011. In 2005, Wideroff et al. [12] found that the awareness level of paternal inheritance of *BRCA 1/2* mutations was 37.5% among the sample physicians. In addition, 34% of those recognized that these mutations exist in less than 10% of breast cancer patients. In 2011, Bellcross et al. [13] examined the awareness and the use of *BRCA 1/2* testing among US primary care physicians. Their study reported that 87% of sampled physicians were aware of breast cancer (BRCA) testing. Although these two studies entail two different aspects of the *BRCA 1/2* topic (one was about mutations, the other was about testing), it still indicates an increase in the general awareness among physicians of *BRCA 1/2* mutations in breast and ovarian cancer that can be detected by *BRCA 1/2* testing. Another genomic testing service identified out of our population of studies is cancer pharmacogenomics (caPGx). caPGx aims to predict responses to cancer treatment therapy and reduce adverse drug reaction based on patients’ genomic variants. A study published in 2014 found that oncologists at a rural site in North Carolina were not well aware of the term “pharmacogenomics” [14]. The overarching theme here shows that there is an increase in awareness knowledge of oncogenomics or basic genomic services in oncology care over time. However, awareness knowledge does not represent a practical or in-depth understanding of how to apply oncogenomics into clinical practices.

### How-to knowledge of applying genomic services in oncology care

With regards to how-to knowledge, the central question concerns whether physicians understand how to apply their oncogenomics knowledge into clinical practice. In other words, the how-to knowledge reflects the level of practice of genomic services in oncology care. This can be measured by the referral rates or the utilization of genomic testing services for cancer patients. How-to knowledge also refers to the ability to provide genetic counselling for cancer susceptibility or the ability to communicate and interpret genomic results to patients. The overall how-to knowledge or the practice level among physicians towards oncogenomics ranges between 20% to 40%. This level of how-to knowledge remains consistent over the period of the fifteen years studied. In a study published in 2003, Wideroff et al. [15] reported that 31% of the sampled U.S. physicians from both primary care and tertiary care had ordered or referred their patients to cancer susceptibility tests (CSTs) or other risk assessment testing. Bellcross et al. [13] found that although 87% of the sampled physicians were aware of *BRCA 1/2* testing, the number of respondents actually ordered at least one test in the past year was only 25%. These findings were consistent with another study published in 2016 that examined the current use of tumor genome sequencing in breast cancer among 215 physicians practicing from 35 countries, 70% of whom are from Europe and 66% work in academic institutions. Gingras et al. [16] reported that 38% of the participants reported having ordered tumor sequencing for breast cancer patients at the minimum level of once in the past. However, Marzuillo et al. [17] found the referral rate of predictive genetic testing for breast cancer and colorectal cancer among a random sample of Italian physicians in the previous two years was only 10% and 5% respectively.

The ability to communicate or interpret genomic results among physicians varies across practitioner specialty and the types of genomic services. Freedman et al. [18] found only 29% of sampled U.S. physicians reported feeling qualified to provide genetic counselling for cancer susceptibility. The researchers also found oncologists tended to feel more qualified than primary care physicians and tertiary care physicians in providing genetic counselling. This finding was consistent with the Dressler et al. [14] study who found 37% of oncologists in their sample were comfortable in interpreting caPGx test results. A recently-published study examined the level of genomic literacy among medical oncologists involved in a cancer clinical genomics trial [1]. Chow-White et al. [1] reported 68% of medical oncologists (MOs) somewhat and strongly agree that they feel confident to communicate genomic results to their patients. The study also showed that half of the MOs (58%) are more confident making treatment decisions after learning about their patient’s genome.

Locations of practice can have an impact on the referral rates of cancer genomic testing services among physicians. Koil et al. [19] reported that physicians in rural areas were less likely to have ordered breast cancer testing than physicians located in urban and suburban areas combined. The how-to knowledge of physicians also varies in terms of the types of genomic testing services. For example, oncologists are more familiar with ordering somatic tests with an average of 100 test orders per year, but much less likely to order germline cancer predisposition tests with an average of 2 test orders per year [20]. The use of caPGx testing also differs depending on the types of cancer tests. Dressler et al. [14] reported that of those oncologists treating breast cancer, 100% used *HER2* gene testing to predict drug response to HERCEPTIN™; 97% used Breast Oncotype DX for chemotherapy treatment decisions, and 32% used *CYP2D2* for drug therapy. In addition, of those treating colorectal cancer, only 26% used Oncotype Dx test for colon cancer treatment decisions. Taken together, although the referral or ordering rates of cancer genomic tests range between 20 and 40% over the fifteen-year period, this how-to knowledge varies by practitioner specialty, location of practice, and the types of genomic services offered. How-to knowledge indicates the usage of genetic testing services, but does not necessarily reflect a deeper understanding of clinical genomics. Greater understanding of clinical genomics or genomic testing could result in lower genomic testing uptake, as physicians are more discrete in ordering or referring to testing, only when they deem it necessary. Hence, a higher level of understanding that impacts the usage or application of clinical genomics is principle knowledge of genomics.

### Principles knowledge of genomics in oncology care

In the case of clinical genomics, Roger’s concept ‘principles of knowledge’ can help us understand fundamental and theoretical concepts of genomic, epigenomic, and transcript alterations in cancer. This can refer to the underlying working mechanism of genetic mutation(s) causing the cancer. Researchers tend to measure principles knowledge of oncogenomics through knowledge tests or self-reported measurements. In knowledge tests, some researchers designed questions concerned with genomic testing services [13, 17] or genetic mutations causing the cancer [12]. For example, in the Bellcross et al. [13] study, the researchers asked participated physicians to select clinical risk assessment scenarios for BRCA testing in accordance with U.S. Preventive Task Force (USPSTF) recommendations. The study shows that only 19% of sampled U.S. physicians identified correctly the high and low risk scenarios indicating the need for BRCA testing. Marzuillo et al. [17] examined the level of knowledge, attitudes and experiences of Italian physicians towards predictive genetic tests, specifically the *BRCA 1/2* and *APC* tests for breast and colorectal cancer. They found that nearly half (43%) of the sample answered correctly all three knowledge questions about *BRCA 1/2* testing, but only 17% responded correctly to *APC* testing. In Wideroff et al.’s study regarding genetic mutations causing breast cancer, only one third of the sample accurately pointed out that *BCRA 1/2* mutations (having a mutation in *BRCA 1/2* genes) exist in fewer than 10% of breast cancer patients [12]. Furthermore, only 13% of the sample accurately responded that colorectal cancer typically carries more than 50% *HNPCC* gene penetrance [12]. The study also found that this type of knowledge varies by specialty – for instance, obstetrics and gynaecology (OBGYN) physicians, medical oncologists, and general surgeons tended to answer more correctly to breast/ovarian questions, as were gastroenterologists to colorectal cancer questions.

Four studies used self-reported measurements of knowledge or confidence scale to examine the principles knowledge of genomics in cancer care. Both Gingras et al. [16] and Gray et al. [21] found a similar level of 21–22% among their samples reported to have low confidence in their genomic knowledge. Using a self-rated knowledge scale, Chow-White et al. [1] reported the majority of MOs in their sample acknowledged to have little knowledge about newer genomic technologies (i.e. high-throughput sequencing genotyping) (50%) and whole genome sequencing (41%). Regarding PGx testing, Dressler et al. [14] pointed out that 33% of oncologists were comfortable with their PGx knowledge. We also identify two factors including locations and years of practice that could influence the principles level of genomic knowledge. Chow-White et al. [1] found that MOs located in urban/suburban areas (73%) tend to have higher genomic literacy than those located in rural areas (27%). Dressler et al. [14] noted that oncologists with less than 10 years of practice (48%) tended to be more comfortable with their PGx knowledge than those with more than 10 years of practice (21%). Overall, the levels of principles knowledge of genomics in oncology care vary among physicians in relation to practitioner specialty, years and location of practice, and the types of genomic services.

### Interventions to provide genomic education for oncology care

#### Overview of the reviewed studies: Methods and interventions

The final and most important aspect of this systematic review is to identify potential interventions to deliver genomic education for oncology care to working physicians. The three categories of knowledge framework dissected the genomic literacy among physicians, which allows us to identify more precise educational interventions of genomics in oncology care. In Table 2, we capture nine studies reporting on the educational interventions along with their methods, outcomes, and limitations. The most common methodology used in these studies is quasi-experimental design, including intervention study, along with pre-and post-knowledge assessment surveys [22–25]. Other studies outline similar methods with a randomized controlled trial of intervention and control group in order to better validate the effectiveness of their intervention [26–30].

The interventions vary by educational theory, content, focus, and delivery length, method and format. Some studies applied an educational outreach theory, known as Kirkpatrick’s framework [27, 28, 31]. The majority of the interventions integrated a variety of delivery formats including in-person training or workshop, online genomic education courses, and online point-of-care genetic resources. Blazer et al. [23] provided training to 131 participants including 48 community-based physicians across the United States in a longitudinal intervention study to improve the use of genetic cancer risk assessment (GCRA) services. The intervention included three stages: “distance didactic learning, face-to-face training, and [twelve] months of web-based professional development activities” [23]. Similarly, a group of researchers in the Netherlands carried out comprehensive oncogenetics training interventions to improve GPs’ genetic consultations skills. The intervention incorporated “an online Continuing Professional Development (G-eCPD) module, a live genetic continuing professional development (CPD) module, and a “GP and genetics” website” as online point-of-care resources providing practical genetic information applicable in clinical practices [25]. In the nine studies included, three were conducted by researchers from the United Kingdom [26, 29, 30], three originated from the same group of researchers in the Netherlands [25, 27, 28], two were from the same group of researchers in the United States [22, 23], and one was carried out as an international collaboration between researchers in Canada and Kenya [24]. Each of these groups developed their educational materials in collaboration with their national or local professional medical societies. The group of researchers from the Netherlands incorporated an oncogenetics eCPD module developed by the Dutch College of General Practitioners [25, 27, 28]. The intervention conducted by researchers from the United States is the result of the curricular and training resources developed by the City of Hope Cancer Genetics Education Program Network funded by National Cancer Institute [22, 23]. These studies show professional medical societies play an important role in designing genomic education programs for physicians to advance the adoption of genomics into clinical care.

#### Outcomes of the interventions

Most of the intervention studies aim to target the awareness, how-to and principles knowledge of oncogenomics among physicians. In the awareness and how-to knowledge, researchers aim to improve the referral rates of genomic services or the genetic consultations skills among physicians. For example, Blazer et al. [22] tested the impact of a cancer genetics education program (CGEP) on improving physicians’ knowledge. Twelve months after the CGEP full day conference, 78% of the respondents among 114 clinician participants reported to have higher level of awareness about cancer genetics. 85% reported to find the information from the CGEP resourceful for their practice. 77% applied the referral guidelines from the program resources to consult genetics risks with their patients, or to order cancer screening tests for their patients. However, the responses in the one-year post-intervention survey were not sufficient to produce convincing evidence on the long-term impact of the intervention. The same group of researchers conducted another study and found the outcomes showed a significant increase in provision of GCRA services (*p* = .018) and high-risk genetic testing referral rates (*p* = .004) after fourteen months of participating in a multi-modal interdisciplinary course [23]. The limitation of this study lies in the bias of their study population. The researchers recruited the majority of participants with former training in oncology or genetics. Hence, this educational intervention may not produce the same outcomes with primary care physicians who do not have training in cancer or genetics.

A group of UK-based researchers found 36 general practices that received educational seminars made 89 referral rates compared to 68 referrals made by general practices that did not receive any intervention [29]. The intervention group also tended to produce higher referral rates (referral rate ratio [RRR] = 1.34) with more accurate cancer referrals (RRR = 2.36). However, the sample size of this study was not adequate to demonstrate anything other than very large changes in clinic attendance rates, and in particular not adequate to demonstrate the effectiveness of the intervention. Different formats of delivery can also produce different impacts on how-to knowledge of physicians. After one year of participating in educational modules on oncogenetics, 68% of those attending the live genetic CPD module reported to be more likely to refer patients to a clinical genetics centre than 29% participating in the online G-eCPD module (*p* < 0.0005) [25]. However, the authors of the study could not validate this finding with the actual clinical genetics referral rates records. A limitation of this study is the self-reporting of long-term genetic consultation skills and referral rates. Also, the number of respondents who participated in the online survey on the genetic competencies and the referral rates was not adequate to demonstrate the statistical power of the data.

Other interventions focus on improving the principles knowledge of oncogenomics. Bethea et al. [26] examined the effects of genetic outreach education program in supporting practitioners’ confidence and skills in handling familial cancers and other genetic conditions. Respondents in the intervention group reported to be more confident in managing patient questions around familial cancers, collecting the relevant family history information, and making better assessment of genetic risks. However, there were only 29 practitioners in the intervention group, which was not sufficient to demonstrate the effectiveness of the outreach program.

In other studies, the outcomes show increases in genomic knowledge after intervention, yet there is no way of gauging how these gains in knowledge might sustain in the long term or translate into applications of genomics in clinical practice. Hill et al. [24] designed an interactive educational workshop for physicians in Kenya in order to increase their childhood eye cancer retinoblastoma genetics knowledge. The participants showed an increase in knowledge post-workshop from 72% to 80%, more particularly increased in knowledge of retinoblastoma causative genetics. However, one year later participants’ knowledge returned to baseline (72%). This showed that the participants needed more frequent reinforcement of the educational intervention. The one-year post-workshop finding of this study is not statistically significant as only 12 respondents took the survey. Houwink et al. [25] tested the long-term impact of online genetics education comprising two-hour online oncogenetics education course, Genetics e-learning Continuing Professional Development (CPD) module. The study yields an increase in knowledge at post-test (0.055 (*P* < 0.05)) and at retention test (six months later) (0.079 (*P* < 0.01)). The respondents also showed high satisfaction with the module. In addition, 90% of the participants reported to apply newly acquired knowledge from the module at least once a month. A small proportion of the participants (5%) frequently used knowledge from the module at the minimum level of once a week. However, the effectiveness of this training in daily practice remains uncertain. The participants remained static on their competencies to self identify disease, refer patients to a specialist, and understand the benefits and risks of genetic testing. Despite high satisfaction in the module and increases in knowledge, the how-to and principles knowledge of oncogenomics and genomic services in oncology care did not change. The limitations of this study lie in the small sample size and self-reported measurements of referral rates.

Wilson et al. [30] evaluated the impact of a multifaceted intervention system to assist general practitioners’ (GPs) confidence and knowledge regarding breast cancer risks. The multifaceted decision-support system is a point-of-care resource developed by GPs in collaboration with clinical geneticists. This system provides educational resources and materials on breast cancer genetics. The intervention and control groups showed no statistically significant difference on their confidence scores. Hence, the intervention was not successful. The intervention’s ineffectiveness may be due to the fact that only a small number of GPs participated in the educational workshop. They might have not also been aware of the decision-supported software and apply it into their clinical practice. In addition, GPs’ confidence in identifying breast cancer genetic risks was a self-reported measurement. Overall, the interventions outlined did not all achieve their goals of improving oncogenomics knowledge among physicians due to many factors such as small sample size, self-reported measurements, or unsustained knowledge gain.

### Factors associated with ordering, referring, or using genomic sequencing

We also focused our analysis on identifying which factors promote an increase in using genomic services in oncology. Six studies in our population identified factors associated with ordering, referring, or using genomic services in oncology practice (Table 3). We categorized these factors into five themes: (1) increases in knowledge, skills, and education, (2) geographical factors, (3) patient interests, requests, and personal health records, (4) professional guidelines, and (5) clinical utility of oncogenomics. Having higher genomic confidence, adequate knowledge of predictive testing for cancer, increasing continuing medical education (CME) or educational materials, and having more time allocated for research would result in physicians wanting to use more genomic services in their practice [15–17, 20].

Geography also plays a role in influencing the use of genomic services. Physicians reported to be more inclined to use genomic services in cancer care in cases where they had greater proximity and access to genomic testing laboratories, and when their practices were located in urban and suburban areas, and especially in the Northeast of the United States [1, 15, 17, 19]. Gingras et al. [16] also identified a statistically significant correlation between more frequent uses of tumor genome sequencing and working in Asia among the MOs (OR 5.76, 95% CI 1.57–21.15, *p* = 0.01). However, this association is questionable as there were only 12 (6%) MOs working in Asia who participated in this study, compared to 151 (70%) MOs working in Europe.

Patient interests and requests for genomic testing is also another significant factor influencing the referral decisions of physicians [17, 19]. Another important determinant for cancer screening referrals is patients’ personal health record or family history of cancer [19]. Furthermore, the availability of professional guidelines for using genomic services endorsed by professional societies such as American Society of Clinical Oncologists (ASCO) would also encourage physicians to use genome services [14, 16]. Regarding the use of cancer pharmacogenomics, there is a clear need for more evidence-based studies or clinical trials demonstrating the clinical utility and effectiveness of the technology [14].

## Discussion

### Knowledge translation of genomics into oncology care

This systematic review provides an insight into the three types of knowledge among physicians towards oncogenomics as well as the outcomes or effectiveness of interventions to provide genomic education for oncology care. We also identify factors associated with the use of genomic services in cancer care among physicians. Genomics is increasingly being adopted in the clinical setting. However, the rapid expansion of genomic research and the clinical uncertainty of the information still engenders doubt, skepticism, and challenges for physicians to fully understand and apply medical genomics into practice [7, 32]. As such, knowledge translation of genomics into oncology care is a slow, thoughtful, and complex process. Our systematic review reveals a number of potential reasons.

We find oncogenomics knowledge among physicians is still limited. We identified an increase in the level of awareness of specific types of oncogenomics such as *BRCA 1/2* mutations and testing [13, 15]. However, there are only three studies in our review that discuss the awareness level of oncogenomics among physicians. This makes it difficult to statistically confirm the precise increase in awareness knowledge. Yet we can assume that the rapid development of genomic research and the increasing number of genomics-related publications improves the general awareness level of knowledge of genomic services in cancer care among physicians. This awareness knowledge also represents the surface level of the complex knowledge regime of genomics. The majority of the studies examining the how-to knowledge of oncogenomics focus on the referral or ordering rates of genomic services. The overall level of referral or ordering rates of genomic services among all the reviewed studies ranges between 20 to 40%. Yet, the how-to knowledge of physicians vary by their specialty [15, 18], locations of practice [19], and the types of genomic services [14, 20]. Due to the nature of provider specialty, ob-gyns are more likely to order *BRCA 1/2* testing, whereas pediatricians are much less likely. A recent study also shows that most pediatric providers of the American Academy of Pediatrics are not comfortable in referring their patients to and consulting them about current genomic services [33]. Pediatric patients are an especially vulnerable population that involve a complex system of ethical, social, and legal issues when it comes to genomic testing. Hence, different provider specialty requires different educational interventions that are tailored to their specialty focus and the characteristics of their patients. The practice level or the how-to knowledge of oncogenomics are key types of knowledge for adoption. However, theoretical or principles knowledge of oncogenomics among physicians can offer more nuanced insight into the missing pieces of the knowledge translation of genomics into oncology care.

If physicians do not understand the nature of genomics and how it applies to clinical practice they may not believe the data innovations and/or act on it. We find that subjective scores of genomic literacy such as self-reported confidence or self-rated knowledge tend to be higher than objective scores of genomic literacy such as scales measuring correct answers to knowledge questions. Both Gingras et al. [16] and Gray et al. [21] found that about 20% of their sampled physicians reported low genomic confidence. That means the majority of the physicians (80%) are confident about their genomic knowledge. In addition, Chow-White et al. [1] noted about half of their sample MOs reported to be knowledgeable about genomic technologies. Yet, two studies using knowledge test scales found that the cancer risk assessment knowledge for *BRCA 1/2* testing and the underlying knowledge about *BRCA 1/2* mutations among physicians in the United States are surprisingly low [12, 13]. Hence, distinguishing between these two principles knowledge constructs would provide a better understanding of how physicians should be educated and which intervention methods should be applied to support physicians in practice. Web-based interventions such as GeneInsight Clinic or Sanford Health Imagenetics Initiative are not genomic educational tools, but point-of-care resources providing support in genomic medicine that can booster physicians’ confidence. Furthermore, similar to how-to knowledge, principles knowledge of physicians vary by their practice specialty [12], locations [1] and years of practice [14], and the types of genomic services [17]. As genomic knowledge tends to be lower with physicians working in rural areas, we suggest detailed attention for future research to both the dissemination of genomic education to these rural areas and their abilities or interests in understanding and adopting genomics in oncology care. These findings provide important clues as to why the knowledge translation of genomics into oncology care is a challenging process, and how it can be improved in the future.

These knowledge assessment studies come with a few limitations. First, the majority of the studies had small sample sizes or a low number of respondents [1, 14, 19–21]. This issue challenges the statistical power and the possibility to generalize the findings. Some studies used self-reported measurements of referral rates, confidence, and knowledge level that make the reliability of the data less credible [18, 20, 21]. Two studies used specialty groups as an independent variable to test for meaningful association [12, 15, 17]. However, the numbers of specialty subgroups participants were not adequate to make any statistically significant comparisons. There are also other issues with response bias [19] and representative nature of the sample [13]. Hence, for future research examining the genomic knowledge among physicians, researchers should pay special attention to the sample size of the population. This is a challenge, since physicians are an elite population that can be reluctant to participate in surveys due to demanding schedules, scarce availability, and high credibility. Researchers should devise strategic plans on how to recruit an adequate number of participants, and how to facilitate an effective process for those participants to volunteer their time. Findings from knowledge test measurements are more reliable than self-reported measurements. However, physicians are a hard-to-reach population. Hence, a survey-based knowledge test could be a demanding request making recruitment very difficult. A work around for measuring referral rates of genomic services could be to check the referral records at the general practices or clinics of physicians.

### Educational interventions to provide genomics training for oncology care

One clear signal from our study is a persistent need for educational interventions to improve genomics knowledge for oncology care. We assessed the outcomes of these interventions in order to pinpoint the most effective strategies to advance the knowledge translation of genomics into oncology practice. The majority of the interventions aimed to improve the principles knowledge of oncogenomics in order to increase the referral rates of genomic services. Hence, in order to evaluate the effectiveness of educational interventions, the researchers evaluated the changes in genomic knowledge or confidence and/or the referral rates. In some studies, researchers also assessed the satisfaction scores among the participants with the interventions. Six of nine studies reported increases in genomic confidence and knowledge of physicians after the interventions [22–24, 26–28]. However, the majority of these studies had low numbers of participants or small sample size. Hence, the effectiveness of these interventions is not statistically significant. Knowledge of genomic topics tended to be subject-specific or designed by different professional medical societies. In some studies, the long-term follow-up effect of the interventions proved to be un-sustained. In addition, some studies used self-reported knowledge or confidence gains, which results in less credibility and reliability of the findings. Three randomized controlled trials studies from the same group of authors reported to find the satisfaction level of their educational interventions to be highly scored [25, 27, 28]. Yet, the sample size of the questionnaires on the satisfaction scores was not adequate to make any statistically significant claim.

Improved genomic confidence or knowledge could potentially lead to higher use of genomic sequencing technologies. Practitioners referral rates of genomic services was reported to improve in four of the nine studies [22, 23, 25, 29]. Yet, some of these studies used self-reported referral rates. This resulted in a mismatch with the actual referral records at clinical genetics centres. Furthermore, higher referral rates do not necessarily mean more accurate or appropriate referrals. Only one study tested the increases in referral rates and the accuracy of the referrals [29]. In order to examine the effects of educational interventions on the how-to knowledge of physicians, researchers should examine the changes of referral rates of the clinics, not of self-reported measurement, and the accuracy of those referrals. Another strategy to assess the effectiveness of the intervention is the applicability of the educational materials in physicians’ daily practice [22, 23, 25, 27, 28]. However, these applicability findings are self-reported measurements. Only one study had empirical data of website visitor analytics to demonstrate that the number of participants visiting the supported genetics website increased [25]. It is challenging to capture the applicability of educational materials in daily practice as the nature of genomics is still full of uncertainty. Through this review, we recognize that many programs have been developed to improve genomic literacy among physicians in cancer care, most of which tailored their interventions with identified competencies and prioritized areas of improvement. Each intervention has its own successes and limitations. From the limitations of all these interventions, we identify an important area of research to study the adoption of genomics in cancer care. We suggest that applying genomics into clinical treatment decisions may require more than just educational guidelines.

As physicians have very limited availability due to heavy workload and time constraints, short-term or long-term interventions could be too cumbersome for practitioners. Increases in genomic knowledge could be a result of long-term exposure to genomic information. Some health educators [34, 35] proposed that ‘just in time’ educational materials or point-of-care resources readily to access by physicians could be an effective way to improve genomic knowledge and assist physicians in using genomics in their daily practice. As genomic research continues to expand exponentially, it is also difficult for educational interventions to keep up with its knowledge expansion. Drawing upon Kirkpatrick’s model [31], the most optimal educational outcome is changes in organizational structures. Our findings also show that increases in genomic knowledge are only one of the determinants for its adoption. Other factors including geography, easy access to genomic testing, patient interests or requests, availability of professional guidelines for genomic testing, and evidence on clinical utility of genomic information are significant indicators for adopting genomics into oncology practice. More evidence-based support from professional medical societies, increased public awareness, and access to genomic testing is necessary organizational changes for the adoption of genomic services in oncology care.

None of the educational intervention studies examined the organizational change and health gain associated with the intervention. Chow-White et al. [1] argued that adopting genomics into oncology care will result in organizational changes in medical practices. The authors observed an ongoing interdisciplinary collaboration between physicians, bioinformaticians, and genome scientists in a clinical cancer genomics trial. Oncologists are still the medical stakeholders that have the medical authority to render treatment decisions for patients. However, oncologists increasingly rely on the expertise of genome data scientists to analyze and interpret the results of genomic information. Therefore, as genomics diffuses into oncology care, it will establish a new regime of clinical systems converging genetics, molecular chemistry, biology engineering, and computational biology. As such, clinical genomics for cancer management will create a new style of oncology practices, bringing together an interdisciplinary set of medical stakeholders. This organizational change in oncology practice is worthwhile to investigate for future research.

## Conclusion

We find the oncogenomic knowledge of physicians across the three types is limited. However, knowledge levels vary by provider specialty, location and years of practice, and types of genomic services. Future research could use Rogers’ knowledge framework to produce a more nuanced analysis of genomic knowledge among practitioners. It is important to understand how genomic information is processed (principles knowledge) and how it influences the treatment decisions of physicians (how-to knowledge). Educational interventions for oncogenomics in this review have shown many limitations in terms of their sustained effects on improving genomic knowledge of physicians and leading to better use of genomic services. There is a dearth of high quality educational interventions that can inform all of the highest outcome level in the Kirkpatrick framework. Future research in educational interventions Future research should attend to improving applications of genomic services in clinical practices along with organizational change engendered by genomics in oncology practice. The limitation of our review is that we did not perform a statistical synthesis of the results due to the small sample and the inconsistency of study design of our reviewed studies. The strength of this study lies in the systematic organization and analysis of the findings of these 21 significant studies into important themes reflecting the three types of knowledge, and the educational interventions related to the adoption of genomic technologies in oncology practice. As genomic science increasingly becomes parts of oncology care, it is critical for genomic literacy to be adopted and increased among practitioners.

## Abbreviations

APC: Activated Protein C Gene
ASCO: American Society of Clinical Oncologists
BRCA 1/2: Breast Cancer Type 1/2 Genes
CaPGx: Cancer Pharmacogenomics
CGEP: Cancer Genetics Education Program. CPD: Continuing Professional Development
CINAHL: Cumulative Index to Nursing and Allied Health Literature
DNA: Deoxyribonucleic acid
ERIC: Education Resource Information Center
GCRA: Genetic Cancer Risk Assessment
G-eCPD: online Continuing Professional Development
GPs: General Practitioners
HGP: Human Genome Project
MOs: Medical Oncologists
PGx: Pharmacogenomics
PRISMA-P: Preferred Reporting Items for Systematic review and Meta-Analysis Protocols
USPSTF: U.S. Preventive Task Force
